# Analysis in a murine model points to IgG responses against the 34k2 salivary proteins from *Aedes albopictus* and *Aedes aegypti* as novel promising candidate markers of host exposure to *Aedes* mosquitoes

**DOI:** 10.1371/journal.pntd.0007806

**Published:** 2019-10-16

**Authors:** Sara Buezo Montero, Paolo Gabrieli, Francesco Severini, Leonardo Picci, Marco Di Luca, Federico Forneris, Luca Facchinelli, Marta Ponzi, Fabrizio Lombardo, Bruno Arcà

**Affiliations:** 1 Department of Public Health and Infectious Diseases, Sapienza University, Rome, Italy; 2 Department of Biology and Biotechnology “L. Spallanzani”, University of Pavia, Italy; 3 Department of Infectious Diseases, Istituto Superiore di Sanità, Rome, Italy; 4 Department of Vector Biology, Liverpool School of Tropical Medicine, Liverpool, United Kingdom; Centers for Disease Control and Prevention, UNITED STATES

## Abstract

**Background:**

*Aedes* mosquitoes are vectors of arboviral diseases of great relevance for public health. The recent outbreaks of dengue, Zika, chikungunya and the rapid worldwide spreading of *Aedes albopictus* emphasize the need for improvement of vector surveillance and control. Host antibody response to mosquito salivary antigens is emerging as a relevant additional tool to directly assess vector-host contact, monitor efficacy of control interventions and evaluate risk of arboviral transmission.

**Methodology/principal findings:**

Groups of four BALB/c mice were immunized by exposure to bites of either *Aedes albopictus* or *Aedes aegypti*. The 34k2 salivary proteins from *Ae*. *albopictus* (al34k2) and *Ae*. *aegypti* (ae34k2) were expressed in recombinant form and *Ae*. *albopictus* salivary peptides were designed through B-cell epitopes prediction software. IgG responses to salivary gland extracts, peptides, al34k2 and ae34k2 were measured in exposed mice. Both al34k2 and ae34k2, with some individual and antigen-specific variation, elicited a clearly detectable antibody response in immunized mice. Remarkably, the two orthologous proteins showed very low level of immune cross-reactivity, suggesting they may eventually be developed as species-specific markers of host exposure. The al34k2 immunogenicity and the limited immune cross-reactivity to ae34k2 were confirmed in a single human donor hyperimmune to *Ae*. *albopictus* saliva.

**Conclusions/significance:**

Our study shows that exposure to bites of *Ae*. *albopictus* or *Ae*. *aegypti* evokes in mice species-specific IgG responses to al34k2 or ae34k2, respectively. Deeper understanding of duration of antibody response and validation in natural conditions of human exposure to *Aedes* mosquitoes are certainly needed. However, our findings point to the al34k2 salivary protein as a promising potential candidate for the development of immunoassays to evaluate human exposure to *Ae*. *albopictus*. This would be a step forward in the establishment of a serological toolbox for the simultaneous assessment of human exposure to *Aedes* vectors and the pathogens they transmit.

## Author summary

Taking advantage of several factors, as worldwide trading, climatic changes and urbanization, *Aedes* mosquitoes are impressively expanding their geographic distribution. A paradigm is provided by the rapid global spreading of *Aedes albopictus*, a species that is a competent vector of several arboviral diseases (e.g. dengue, Zika, chikungunya) and has been responsible of quite a few outbreaks in the last decade. Historically, vector control always played a pivotal role for the containment of arthropod-borne diseases, and this appears especially crucial for arboviral diseases for which no effective vaccines or specific medications are available. Currently, host exposure to mosquitoes is indirectly evaluated by entomological methods; however, exploitation of human immune responses to mosquito salivary proteins is emerging as a relevant additional tool, with important epidemiological implications for the evaluation of mosquito-borne disease risk. This study provides preliminary but solid indications that the 34k2 salivary proteins from *Ae*. *albopictus* and *Aedes aegypti* may be suitable candidates for the development of serological assays to evaluate spatial and/or temporal variation of human exposure to *Aedes* vectors. Combined to the presently available tools to assess arboviral exposure/infection, this may be of great help for the development of a serological toolbox allowing for the simultaneous determination of human exposure to *Aedes* vectors and to the pathogens they transmit.

## Introduction

In the last decades *Aedes* mosquitoes have been responsible for an increased transmission and severe outbreaks of arboviral diseases as dengue, chikungunya, Zika and yellow fever, creating a renewed challenge for public health. Dengue viruses (DENV), with a nearly ubiquitous distribution in the tropics, may be responsible for more than 100 million symptomatic infections and over 20,000 deaths per year [1]. Zika virus (ZIKAV), which became widely known in 2015 after the epidemic emergence in Brazil, caused ~500,000 cases in 2016 and its transmission is currently ongoing in at least 61 countries, mostly in the Americas but also in Western Pacific, Africa and Southeast Asia [2, 3]. Chikungunya virus (CHIKV), after the major outbreak in Reunion Island in 2005 [4], has caused additional epidemics in both tropical and temperate regions of the world, with a very large one in 2015–2016 involving over 1 million suspected cases in the Americas [5, 6]. Even the yellow fever virus (YFV), for which a safe and effective vaccine is available since decades, and whose transmission has been in decline for several years, is currently endemic in 47 countries in Africa and Central/South America, and a modelling study estimated a disease burden of at least 85,000 cases and 30,000 deaths in 2013 [7, 8]. The main vector of these arboviruses is *Aedes aegypti*, however the tiger mosquito *Aedes albopictus* is gaining increasing attention due to its very rapid worldwide spreading and its vector competence [9, 10]. In fact, *Ae*. *albopictus* can act as epidemic driver in areas where *Ae*. *aegypti* is absent or present at low levels, as testified for example by the chikungunya outbreak in Reunion Island in 2005 [4] or by the several cases of autochthonous transmission of CHIKV and DENV recorded in Italy, France and Croatia from 2007 to 2018 [11]. Moreover, the appearance of viral mutations significantly enhancing adaptation to vectors [12, 13] and the geographical spread of both these vector species due to globalization [14] are raising growing concern in public health authorities. To date no specific drugs can be employed to treat human cases. A dengue vaccine has recently been licensed but its use is recommended only for individuals with known prior DENV infection [15], and modelling studies predict achievement of cost-effectiveness only in high-transmission areas of dengue-endemic countries [16]. Therefore, the main method to limit the transmission of these arboviral diseases is still to control mosquito vector populations and prevent their contact with humans.

The evaluation of human exposure to *Aedes* mosquitoes, which is of great importance to assess arboviral transmission risk and guide vector control strategies, is currently based on entomological measures that provide estimates of immature and/or adult mosquito densities in a defined area [17]. However, entomological indices yield an indirect assessment of human-vector contact, are labor-intensive, costly, difficult to implement in some epidemiological settings (e.g. low vector density) and can be applied at the community level only. Progress made in the last fifteen years in the understanding of composition and complexity of blood feeding arthropod saliva paved the way toward the development of novel complementary tools to directly evaluate human exposure to disease vectors, with interesting implications for the improvement of vector control and prediction of disease risk. In fact, while feeding on their hosts, blood sucking arthropods inject a cocktail of salivary proteins whose main role is to allow for an efficient blood meal by inhibiting host hemostatic and inflammatory responses [18]. Independently from its physiological role, saliva of blood feeders also evokes in vertebrates an antibody response that can be exploited to evaluate exposure to disease vectors; this concept was first proposed/shown for ticks [19] and then extended to several other blood feeding arthropods including anopheline and culicine mosquitoes [20–22]. However, using mosquito saliva as antigen for immunoassays is largely impracticable for several reasons. First, obtaining large amounts of saliva or salivary gland extracts (SGE) is laborious and time-consuming. Second, reproducibility may be a problem, both because saliva composition may vary according to mosquito physiological states and due to technical variations in the procedure of saliva collection or SGE preparation. Finally, and most importantly, saliva is a mixture of several dozen salivary proteins, some of which are widely spread among blood feeding arthropods, and this may give rise to potential problems of cross-reactivity both at the genus and eventually even at family level. However, the large amount of information made available by transcriptome studies on salivary protein repertoires of blood feeding insects [23] highlighted the existence of several family- and genus-specific salivary proteins, which may represent ideal candidates as markers of host exposure to specific disease vectors.

Within the family *Culicidae*, groups of anopheline- and culicine-specific salivary proteins have already been identified [24, 25] and a clear proof of concept has been provided for the gSG6 salivary protein from *Anopheles gambiae*. In fact, the gSG6 protein or the gSG6-P1 peptide have been validated as markers of human exposure to malaria vectors in a large variety of epidemiological settings in Africa [26–31]; in addition, evidence of their possible utility to evaluate exposure to Asian [32] and Polynesian [33] malaria vectors has been more recently obtained. So far an equally well established and widely validated marker is not available for *Aedes* mosquitoes, even though very promising indications came by the exploitation of the Nterm-34kDa peptide, which is designed on the culicine-specific 34k1 salivary protein from *Ae*. *aegypti* (reviewed in [34]). Studies in exposed children from different villages in Benin [35] and in Côte d’Ivoire [36], as well as a retrospective study on a population from Laos exposed to DENV [37], suggested that the Nterm-34kDa peptide may allow to detect variation in human exposure to *Ae*. *aegypti* bites. Moreover, the IgG response to the Nterm-34kDa peptide has been employed to assess vector control implementation in an urban area at La Reunion Island, where individuals were exposed to *Ae*. *albopictus* and not to *Ae*. *aegypti*. As a consequence, it has been proposed that the IgG antibody response to the *Ae*. *aegypti* Nterm-34kDa salivary peptide may be a relevant short term indicator to evaluate the efficacy of vector control interventions against *Aedes* mosquito species [38].

Previous studies indicated that human antibody responses to mosquito salivary proteins are heterogeneous, with some individuals responding to one antigen but not to others and with different salivary proteins eliciting IgG responses that are quantitatively and qualitatively diverse [29]. In this respect, the availability of more than a single salivary antigen may be very useful, especially in different epidemiological settings (e.g. high versus low mosquito density), providing a better view of human exposure to *Aedes* vectors and disease risk, and eventually increasing the sensitivity and/or specificity of the immunoassays. Moreover, although the *Ae*. *aegypti* Nterm-34kDa peptide was successfully used to evaluate exposure to *Ae*. *albopictus* [38], the N-terminal region of the 34k1 protein is relatively divergent in these two species (12 identical residues over 19 with a 3 amino acids gap), suggesting that the availability of markers based on *Ae*. *albopictus* salivary proteins may provide some advantages. In the present study the suitability of novel candidate salivary markers of host exposure to *Aedes* mosquitoes was evaluated in an experimental model where mice were subjected to a controlled regimen of exposure to bites of *Ae*. *albopictus* or *Ae*. *aegypti*. In addition, an hyperimmune serum from a human volunteer was used to provide some preliminary but valuable indication on the antigenicity to humans of the recombinant 34k2 salivary protein from *Ae*. *albopictus*.

## Methods

### Ethical statement

According to D.lgs 26/2014, which has transposed in Italy the European Directive 2010/63/EU on the protection of animals used for scientific purposes, the animal research protocol has been reviewed and approved by the Animal Welfare Body of the Istituto Superiore di Sanità (Italian National Institute of Health) and authorized by the Italian Ministry of Health with authorization number 150/2016-PR of 19^th^ February 2016. The human serum employed in this study was provided from a single donor who, for his own purposes (colony maintenance) and independently from this study, had regularly fed an *Ae*. *albopictus* colony. This hyperimmune healthy adult donor provided written informed consent for the use of the serum to measure IgG antibody levels against mosquito salivary proteins. No formal request for approval on the use of this serum, which was provided by the hyperimmune donor on a pure voluntary basis, was submitted to the authors’ institutional review board or equivalent committee.

### Mosquito rearing and salivary gland extracts preparation

*Ae*. *albopictus* (originally collected in Rome, Italy) and *Ae*. *aegypti* (originally collected in Reynosa, Mexico) were reared in the insectary of Sapienza University of Rome and Istituto Superiore di Sanità under standard conditions (27 ± 1°C, 70% relative humidity, 14:10 hours light:dark photoperiod) and colony maintenance achieved by feeding on guinea pigs or by membrane feeding using rabbit blood. Adult female mosquitoes 3–8 days post-emergence (dpe), and never fed on blood before, were used for all the experiments. Mosquitoes were starved for at least 6–8 hours before exposure to mice. Salivary glands were dissected in Phosphate Buffered Saline (PBS), transferred into a tube containing 20 μl of PBS and frozen at -80°C in batches of 20–40 salivary glands. Salivary gland extracts (SGE) were prepared by three cycles of freezing and thawing followed by centrifugation at 16,000 x g at 4°C. Supernatants were collected and protein concentration measured by the Bradford method (Bio-Rad Laboratories Inc., USA) after pooling the different batches in order to generate a homogeneous SGE stock to be used for all ELISA assays. Protein concentration was determined using the Take3 micro-volume plate in a BioTek microplate reader (BioTek Synergy HT). SGE stocks were aliquoted and stored at -20°C until use.

### Mice immunization and sera collection

Female BALB/c mice, aged 6–8 weeks were obtained from Charles River Laboratories and kept in the animal facility of Istituto Superiore di Sanità according to approved Institutional Animal Care and Use Committee protocols. Two cohorts, composed of 4 naïve mice each, were anesthetized and exposed to bites of either *Ae*. *albopictus* or *Ae*. *aegypti*. Briefly, the abdomen of each mice was exposed for ~20 minutes to one of four paper cups covered with a mesh net containing 33–47 adult female mosquitoes (either *Ae*. *albopictus* or *Ae*. *aegypti*) per mice. All mice were exposed on the same day every 2 weeks for 6 weeks (total 4 times), an exposure regimen similar to those previously employed for immunization to anopheline mosquito saliva [39, 40]. The number of mosquitoes who successfully fed on each mouse following each exposure is reported in Supplementary S1 Table. An additional group of mice not exposed to any mosquito was also included in the experimental plan as a further negative control. Small blood aliquots (~50–100 μl) were collected from the tail vein for serum preparation at different time points: one week before the 1^st^ exposure (B, baseline), one week after the 2^nd^ exposure (M, midterm), one week after the 4^th^/last exposure (T, top) and then 1, 2 and 3 months after the end of the exposure regimen (+30, +60 and +90, respectively). Finally, 5 months after the last exposure (+150) mice were sacrificed and larger blood volumes (> 600 μl) collected by cardiac puncture. After blood clotting sera were separated by centrifugation at 10,000 g for 15 minutes and stored at -20°C.

### Human hyperimmune serum

An hyperimmune serum was obtained in February 2013 from a volunteer who had been regularly feeding, for his own purposes (colony maintenance) and independently from this study, an *Ae*. *albopictus* colony fortnightly in the previous 4 months. Thirty-nine months later, in May 2016, a second serum aliquot was obtained from the same donor who had not been feeding *Ae*. *albopictus* nor other *Aedes* spp colonies for at least twenty-four months and had eventually only natural exposure to *Aedes* mosquitoes. Written informed consent for participation to this study was provided from the volunteer.

### Peptide design

Peptides were designed on *Ae*. *albopictus* salivary proteins previously identified as restricted to culicine mosquitoes [25, 41, 42] and exhibiting limited amino acid identity (<50%) to *Culex* species. Potentially immunogenic peptides were selected using four different bioinformatic tools for the prediction of B-cell epitopes and immunogenic regions: BepiPred [43], ABCpred [44], Bcepred [45] and Epitopia [46, 47]. Five peptides 21–23 amino acids in length were designed on three *Ae*. *albopictus* salivary proteins and chemically synthesized by Biomatik Corporation (Canada): alb34k1-P1 (HPLPEEATSDAAIKCTLSEED), representing the N-terminus of the 34k1 protein (AAV90689); alb34k2-P2 (TVSEEDLTTIRNAIQKASRASLD) and alb34k2-P3 (ALKFYPKTGNKEANEADIRGRQF), designed in the N- and C-terminal regions of the 34k2 salivary protein (AAV90690); alb62k1-P4 (LTHIEKPIYTEEAESETSDSDE) and alb62k1-P5 (YGLSGMRSGGIPDNHAEWKLNA) designed in the N- and C-terminal regions of the 62k1 protein (AAV90683).

### Expression and purification of the *Ae*. *albopictus* and *Ae*. *aegypti* 34k2 salivary proteins

The sequence encoding for the mature *Ae*. *albopictus* 34k2 protein (mRNA AY826118, protein AAV90690) was obtained by cDNA synthesis followed by PCR amplification. Briefly, total RNA was extracted from salivary glands of *Ae*. *albopictus* females (6 dpe) using the TRIzol reagent (ThermoFisher Scientific) and cDNA synthetized by the iScript cDNA synthesis kit (Bio-Rad). The region encoding for the mature protein was amplified using the high-fidelity Platinum Pfx DNA polymerase (ThermoFisher Scientific) and the primers 5’-AGTCGGATCCAACCCAACCCCAAAGTCG-3’ (forward) and 5’-CGTAGCGGCCGCTATTACAATGTACCCCTTAAGCCC-3’ (reverse) carrying *Bam*H I and *Not* I restriction sites. The PCR product was first cloned into the PCRII TOPO TA vector (ThermoFisher Scientific) and then directionally subcloned into a modified pETSUMO vector (Invitrogen), which allows for the expression of recombinant proteins fused at their N-terminus to a 8xHis-tag and SUMO protein in order to help purification and increase solubility, respectively [48]. The sequence encoding the mature *Ae*. *aegypti* 34k-2 protein (mRNA AF466595, protein AAL76018) was purchased from GENEWIZ UK as synthetic gene, codon-optimized for *Escherichia coli* expression, and subcloned into the modified pETSUMO vector. Recombinant proteins were expressed in the T7 SHuffle *E*. *coli* K12 strain (New England Biolabs). Pre-cultures from a single colony were grown overnight at 30°C in 100 ml of LB medium supplemented with 50 μg/ml kanamycin in a 500 ml flask. One liter of preparative scale cultures in autoinducing medium ZYP5052 [49] were inoculated with 50 mL of the overnight pre-culture and grown at 30°C for 4.5 hours. The temperature was then set at 20°C and cultures were let grow overnight. Cells were harvested at 3000 *g*, resuspended in buffer A (25 mM HEPES, 500 mM NaCl, pH 8.0) and lysed by sonication. The cleared lysate, obtained after centrifugation at 75000 *g* for 45 minutes at 4°C, was loaded onto a HisTrap excel column (GE Healthcare) using an Äkta system (GE Healthcare) at room temperature. Protein elution was performed with buffer B (25 mM HEPES, 500 mM NaCl, 250 mM imidazole, pH 8.0). The eluted sample was incubated with His-tagged SUMO protease and dialyzed overnight in buffer A at 4°C. After removal of His-SUMO tag and His-SUMO protease through a second passage on the HisTrap column, the sample was concentrated using Vivaspin Turbo 15 filters (Sartorius, MWCO 10 kDa) and loaded onto a Superdex 75 10/300 GL column equilibrated in 25 mM HEPES, 100 mM NaCl, pH 8.0. Protein concentration was evaluated determining the absorbance at 280 nm and assuming, according to the Expasy ProtParam tool [50], extinction coefficients of 0.82 and 0.84 for the *Ae*. *albopictus* and the *Ae*. *aegypti* protein, respectively. Purified proteins were concentrated and stored at -80°C until usage.

### Enzyme-linked immunosorbent assays and data analysis

Enzyme-linked immunosorbent assays (ELISA) were essentially performed as previously described for the *An*. *gambiae* gSG6 protein [51]. Briefly, coating was performed in 50μl of Coating Buffer (15mM Na_2_CO_3_, 35mM NaHCO_3_, 3mM NaN_3,_ pH 9.6) for 3 hours at room temperature in 96-well plates (Nunc Maxisorp) using 20 μg/ml of peptides or 5μg/ml of purified recombinant proteins (al34k2 and ae34k2). Salivary gland extracts (SGE) were used at a concentration of 10 SG/ml (i.e. at a protein amount per ml equivalent to 10 salivary glands), which corresponded to 8.6 μg/ml for *Ae*. *albopictus* and 11.0 μg/ml for *Ae*. *aegypti*. Plates were: (i) blocked for 3 hours at room temperature (150 μl 1% w/v skimmed dry milk in PBST); (ii) incubated overnight at 4°C with 50μl of a 1:50 dilution of sera; (iii) incubated for 3 hours at room temperature with 100μl of a goat anti-mouse IgG horseradish peroxidase-conjugated antibody (Pierce 31430, 1:10000 dilution); (iv) incubated in the dark at 25°C for 15 minutes with 100μl of o-phenylenediamine dihydrochloride (OPD, Sigma P8287) for colorimetric development. Reactions were terminated by adding 25μl of 2M H_2_SO_4_. Three to four washings were performed between each step. OD_492_ were determined using a microplate reader (Biotek Synergy HT). All samples were analyzed in duplicate with the antigen and once with no antigen. The no antigen well was used for background subtraction and results were expressed as ΔOD values, which were calculated according to the formula ΔOD = ODX−OD_N_, where OD_X_ represents the mean of the duplicate with the antigen and OD_N_ the value in the well without antigen. Negative and positive controls were included to control for intra- and inter-assay variation, which was always below 20%. Graph Pad Prism Software (San Diego, CA USA) was used for graph preparation.

The tentative estimation of cross-reactivity was made taking into account ΔOD levels measured in mice sera at four different time points: T (fully developed response), +30, +60 and +90 (possible start of declining). For each *Ae*. *albopictus*-exposed mouse and time point the ratio between the IgG responses to aeSGE and alSGE was considered, and the mean values among the four mice for each time point calculated. The mean value among the four time points was 0.60 (range 0.55–0.64). A similar calculation was made for *Ae*. *aegypti*-exposed mice considering the ratio between ΔOD levels alSGE/aeSGE, which yielded a mean value among the four time points of 0.41 (range 0.34–0.47). Combining these results a rough provisional estimation of the level of immune cross-reactivity of SGE from these two species in our experimental mice could be of approximately 50%.

## Results

### Mice immunization by exposure to *Ae*. *albopictus* or *Ae*. *aegypti*

To evaluate the immunogenicity of candidate peptides and recombinant proteins we first immunized groups of 4 naïve BALB/c mice by exposure to bites of *Ae*. *albopictus* or *Ae*. *aegypti*. Overall, blood feeding efficiency was unexpectedly higher for *Ae*. *aegypti* (80.6%) than for *Ae*. *albopictus* (49.6%), with an average of 28 and 21 fed mosquitoes/mouse/exposure, respectively (Table 1). Small blood aliquots (~50–100 μl) were collected at different time points as described in the Method section.

**Table 1:** Mean number of mosquitoes and percentage feeding.

	mosquito n	fed n	fed %
*Ae*. *albopictus*	44 (41.5–47.0)	21 (17.8–24.0)	49.6 (42.6–57.8)
*Ae*. *aegypti*	35 (33.0–37.5)	28 (25.5–30.3)	80.6 (74.1–86.8)

Number of mosquitoes and percentages represent the mean per mouse per exposure. Ranges are in brackets.

**Fig 1:**
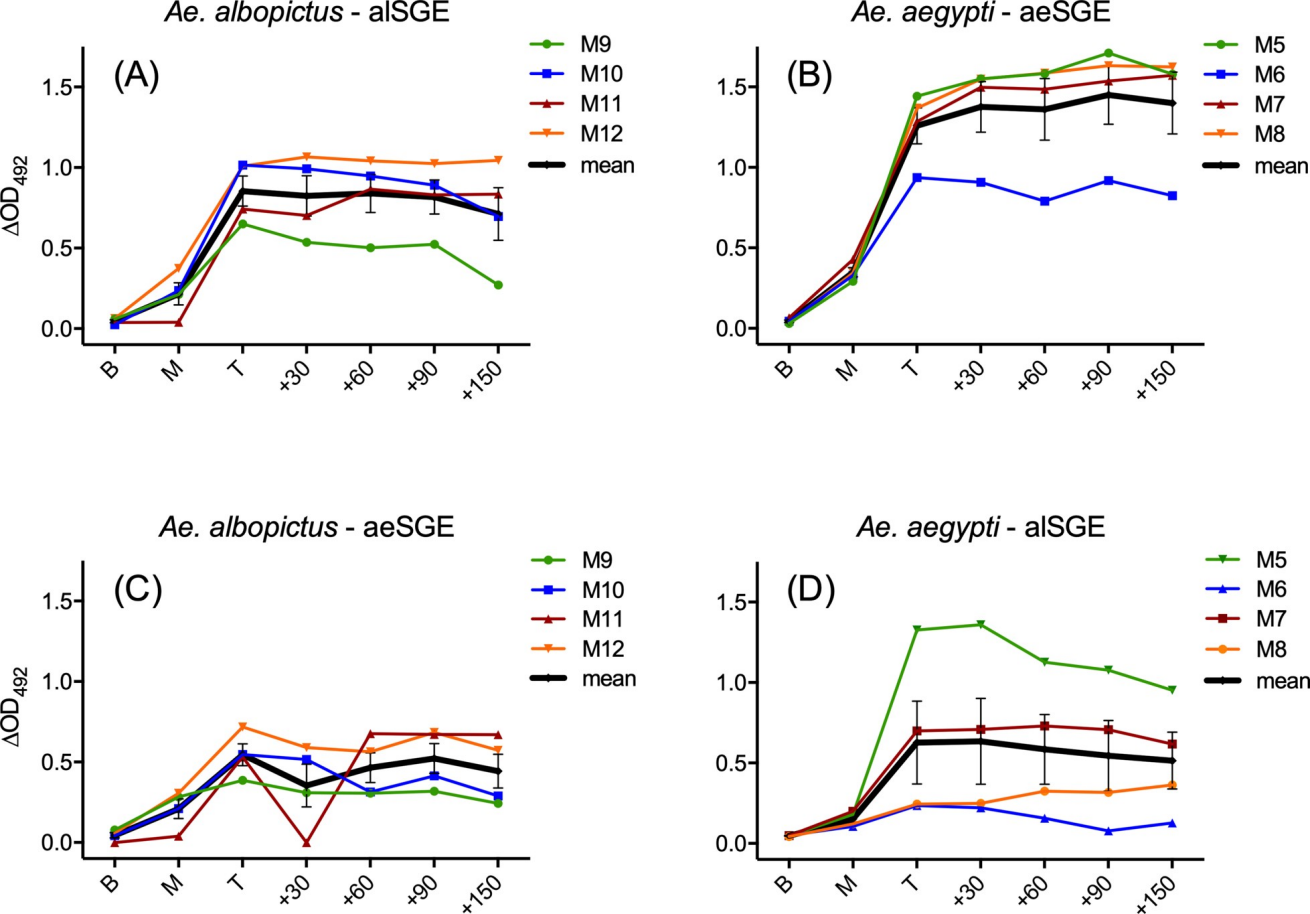
Anti-SGE IgG responses of mice exposed to bites of either *Ae*. *albopictus* or *Ae*. *aegypti*. IgG responses of *Ae*. *albopictus*-exposed mice to SGE from *Ae*. *albopictus* (alSGE) and from *Ae*. *aegypti* (aeSGE) are shown in panels A and C, respectively. IgG responses of *Ae*. *aegypti*-exposed mice to aeSGE and alSGE are reported in panels B and D. IgG levels are expressed as ΔOD values at 492 nm. The response of the individual mice is in color as reported in the legends. Thick black lines represent mean ΔOD values, bars denote standard errors. The different time points are as follows: B = baseline, one week before exposure; M = midterm, one week after the second exposure; T = top, one week after the fourth and last exposure; +30/+60/+90/+150, 30/60/90/150 days post-exposure.

Mice immunization was verified measuring by ELISA the IgG responses to salivary gland extracts (SGE) of the corresponding mosquito species. All mice exposed to *Ae*. *albopictus* developed an antibody response to alSGE, with anti-saliva IgG levels increasing after the second exposure, reaching a peak one week after the fourth/last exposure and remaining essentially unchanged up to 3–5 months post-exposure (Fig 1A). A similar pattern was found in mice exposed to *Ae*. *aegypti*, even though IgG levels against aeSGE appeared higher in most mice (Fig 1B). Overall, independently from inter-individual and inter-species quantitative differences, these observations indicate that the exposure regimen was effective both for *Ae*. *aegypti* and *Ae*. *albopictus*, with all mice developing anti-SGE IgG responses.

The salivary proteins of *Ae*. *albopictus* and *Ae*. *aegypti* were estimated to share, on average, ~70% amino acid identity [41]; therefore, we wondered if mice exposed to *Ae*. *albopictus* could recognize aeSGE and vice versa. Not surprisingly, IgG raised by exposure to saliva of one species could recognize SGE from the other species (Fig 1C and 1D), indicating a certain degree of cross-reactivity due to the common and relatively conserved repertoire of salivary proteins [23, 41, 42].

### Selection of candidate *Ae*. *albopictus* salivary proteins and peptide design

As a first approach toward the identification of candidate salivary antigens for the development of immunoassays to evaluate host exposure to *Ae*. *albopictus* we decided to try the design of peptides, which could be tested using sera from the immunized mice. Noteworthy, peptides designed on mosquito salivary proteins, namely the gSG6-P1 and the Nterm-34kDa peptides, have been already successfully used to assess human exposure to *Anopheles* [27, 52–54] and *Aedes* vectors [35, 38], respectively. Therefore, a group of *Ae*. *albopictus* salivary proteins were selected (i) on the basis of culicine-specificity, i.e. their absence in the saliva of anophelines or other blood feeding arthropods [25, 41, 42], (ii) according to their limited identity (< 50%) to homologs from *Culex* species and (iii) taking also into account previous indications of immunogenicity [55]. Considering only peptides whose antigenicity was predicted by multiple tools we ended up with five candidates from three different *Ae*. *albopictus* proteins: 34k1 (AAV90689) and 34k2 (AAV90690), both members of the 34 kDa salivary protein family, and the 62k1(AAV90683) protein, belonging to the 62 kDa family. Members of both these protein families are found exclusively in *Aedes* mosquitoes, are highly enriched or specifically found in adult female salivary glands [41, 42] and, notably, were previously shown to be immunogenic to humans [55]. The physiological role of the 34 kDa and 62 kDa salivary proteins in blood feeding is presently unknown, however the 34k1 *Ae*. *aegypti* protein was found to enhance DENV replication in human keratinocytes [56] and its silencing in the mosquito by RNAi reduced DENV2 replication in the salivary glands [57]. The first peptide, alb34k1-P1 (21 amino acids) is designed on the N-terminus of the *Ae*. *albopictus* 34k1 protein in a position corresponding to the Nterm-34kDa salivary peptide (19 amino acids) designed on the *Ae*. *aegypti* ortholog [35]. The four remaining peptides alb34k2-P2, alb34k2-P3, alb62k1-P4 and alb62k1-P5 were designed in the N- and C-terminal regions of the 34k2 and 62k1 salivary proteins, respectively.

IgG responses to these peptides were analyzed by ELISA in mice immunized to *Ae*. *albopictus* or *Ae*. *aegypti* saliva. However, even using low sera dilutions (1:20) and high peptide concentrations (20 μg/ml), and also mixing together the five peptides, no response was observed in any mice. IgG responses to the peptides were also analyzed in the same conditions using a human serum from a donor hyperimmune to *Ae*. *albopictus* saliva but no IgG recognizing the peptides could be revealed.

### Expression of recombinant 34k2 salivary proteins from *Ae*. *albopictus* and *Ae*. *aegypti*

**Fig 2:**
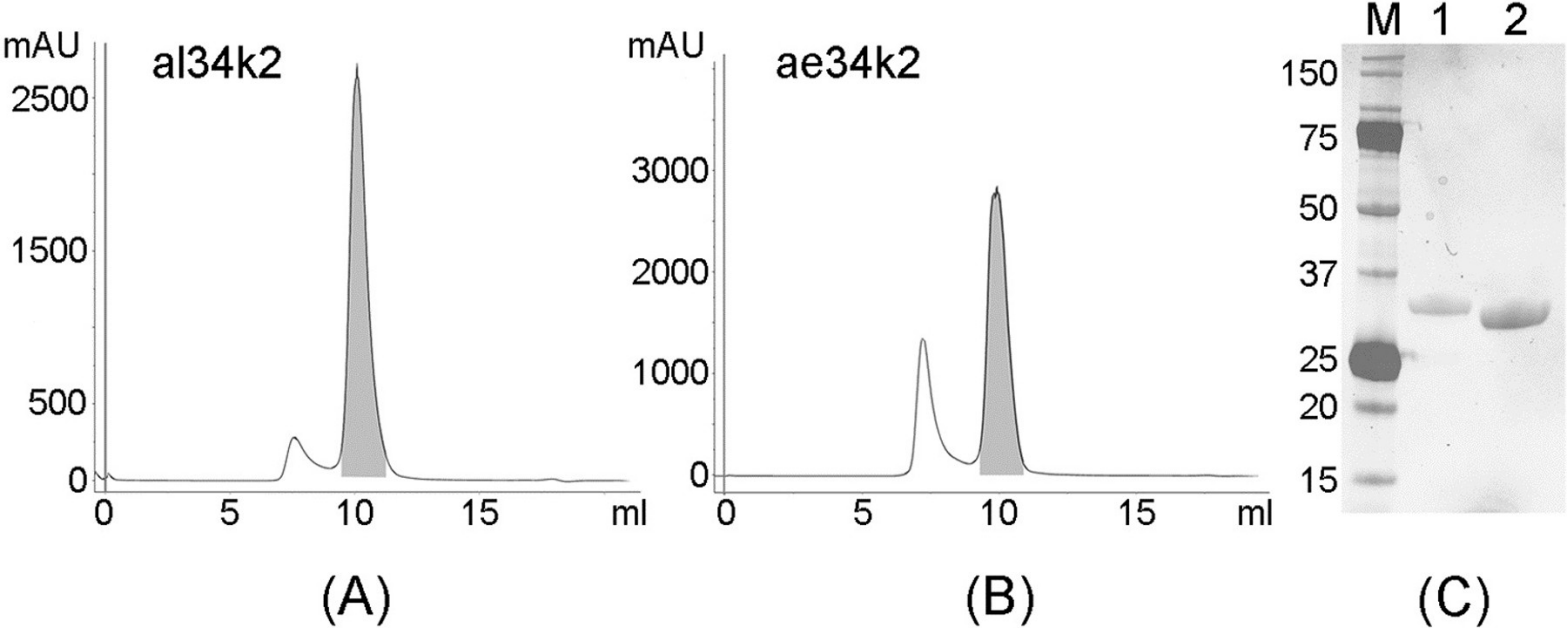
Purification of the *Ae*. *albopictus* and *Ae*. *aegypti* 34k2 recombinant proteins. Size exclusion chromatograms (Superdex-75 10/300 GL, GE Healthcare) showing the peaks (shaded) corresponding to the pure *Ae*. *albopictus* (A) and *Ae*. *aegypti* (B) 34k2 salivary proteins. Fractions corresponding to the peaks were pooled, analysed by SDS-PAGE on a Mini-Protean TGX Stain-free precast gel in non-reducing conditions and stained with Coomassie Brilliant Blue R-250 (C). M, Molecular Weight Marker; 1, al34k2; 2, ae34k2.

As a second parallel approach, the expression in recombinant form of a few candidate salivary proteins from *Ae*. *albopictus*, including the 62k1 and 62k2 proteins, was attempted. Specifically, conditions for expression and purification of the *Ae*. *albopictus* 34k2 salivary protein, for which previous indication of immunogenicity were available [55], were optimized. The 34 kDa family of salivary proteins was originally identified in *Ae*. *aegypti* and found to be composed by at least three members, two of which, named 34k1 (ABF18170) and 34k2 (AL76018), are abundant in saliva and enriched or specifically expressed in adult female salivary glands [42]. Two family members, orthologs of the *Ae*. *aegypti* 34k1 and 34k2 and with a similar expression profile, were found in *Ae*. *albopictus* [41]. Among Metazoan, proteins of the 34kDa family are only found in culicine mosquitoes and, due to the intron less nature of their genes, it has been suggested they may have been acquired by horizontal transmission. Orthologs between the two *Aedes* species share 65% (34k1) and 62% (34k2) amino acid identity, whereas paralogs exhibit 32–33% identity. Members of the 34kDa family appear to be present also in *Culex* species [58, 59], however they are only distantly related to the *Aedes* proteins (23% to 28% identity). We will refer to the *Ae*. *albopictus* and *Ae*. *aegypti* 34kDa proteins as al34k1/al34k2 and ae34k1/ae34k2, respectively. The al34k2 protein was successfully expressed in *E*.*coli* and purified to homogeneity and, after some initial tests indicating its immunogenicity, also the ae34k2 salivary protein from *Ae*. *aegypti* was expressed/purified in a similar manner (Fig 2).

### IgG responses to the *Ae*. *albopictus* and *Ae*. *aegypti* 34k2 salivary proteins

**Fig 3:**
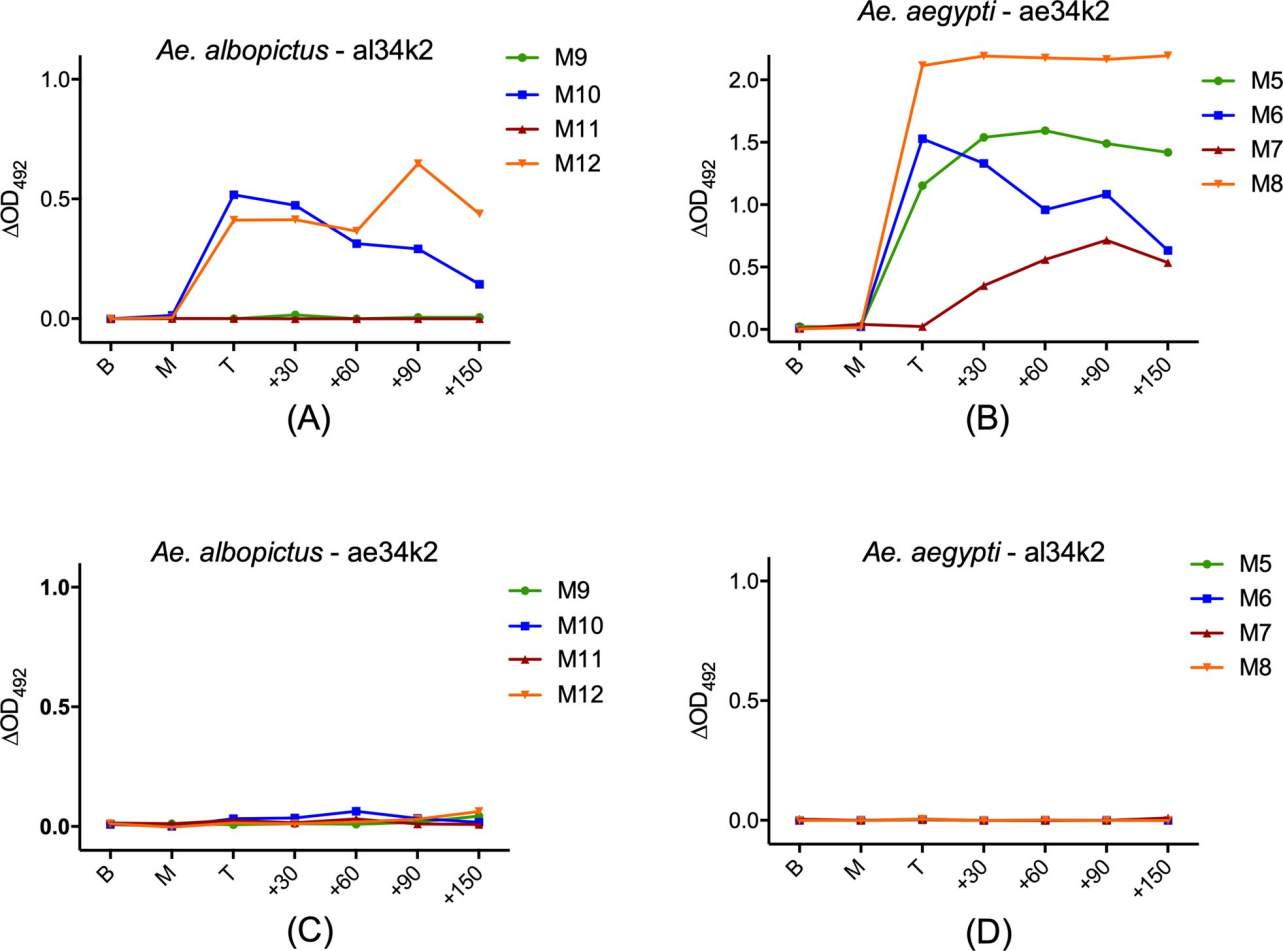
IgG responses to al34k2 and ae34k2 of *Ae*. *albopictus*- and *Ae*. *aegypti*-exposed mice. Anti-al34k2 (A) and anti-ae34k2 (C) IgG levels in *Ae*. *albopictus*-exposed mice. IgG responses of *Ae*. *aegypti*-exposed mice to ae34k2 and al34k2 are shown in (B) and (D), respectively. Time points as in Fig 1.

IgG antibody levels against the al34k2 and ae34k2 were measured by ELISA in mice exposed to bites of *Ae*. *albopictus* or *Ae*. *aegypti*, respectively. Two out of four *Ae*. *albopictus*-exposed mice (M10 and M12) showed IgG responses to al34k2. In both mice the response reached a peak one week after the last exposure and was stable up to 2 months after the end of the exposure regimen. The response then decreased gradually in M10 and, instead, persisted or even had some increase in M12. No anti-al34k2 IgG responses were detectable in the other two mice (M9 and M11) at any time point (Fig 3A). As far as the *Ae*. *aegypti*-exposed mice are concerned, all mice exhibited IgG responses to ae34k2, although at a different degree and with slightly different kinetics (Fig 3B). The response reached a peak one week to one month after the last exposure and then stayed unchanged in M5 and M8, continued to slightly increase in M7 and showed a trend to decrease in M6. These results indicate that, despite some inter-individual variability, both al34k2 and ae34k2 are immunogenic to mice. The higher IgG levels and the responses of all *Ae*. *aegypti*-exposed mice may be due to the more effective immunization to saliva achieved in these mice (likely because of the higher number of bites/mouse/exposure) as also indicated by the IgG responses to SGE (Fig 1). Interestingly, considering the relatively high conservation of the 34k2 proteins in the two *Aedes* species, no immune cross-reaction was observed. Indeed, IgG antibodies directed against al34k2 could not recognize the *Ae*. *aegypti* protein and, conversely, anti-ae34k2 IgG did not recognize the *Ae*. *albopictus* protein (Fig 3C and 3D). These observations suggest that the 34k2 proteins from *Ae*. *aegypti* and *Ae*. *albopictus* may represent interesting species-specific markers to evaluate host exposure to these two *Aedes* species.

**Fig 4:**
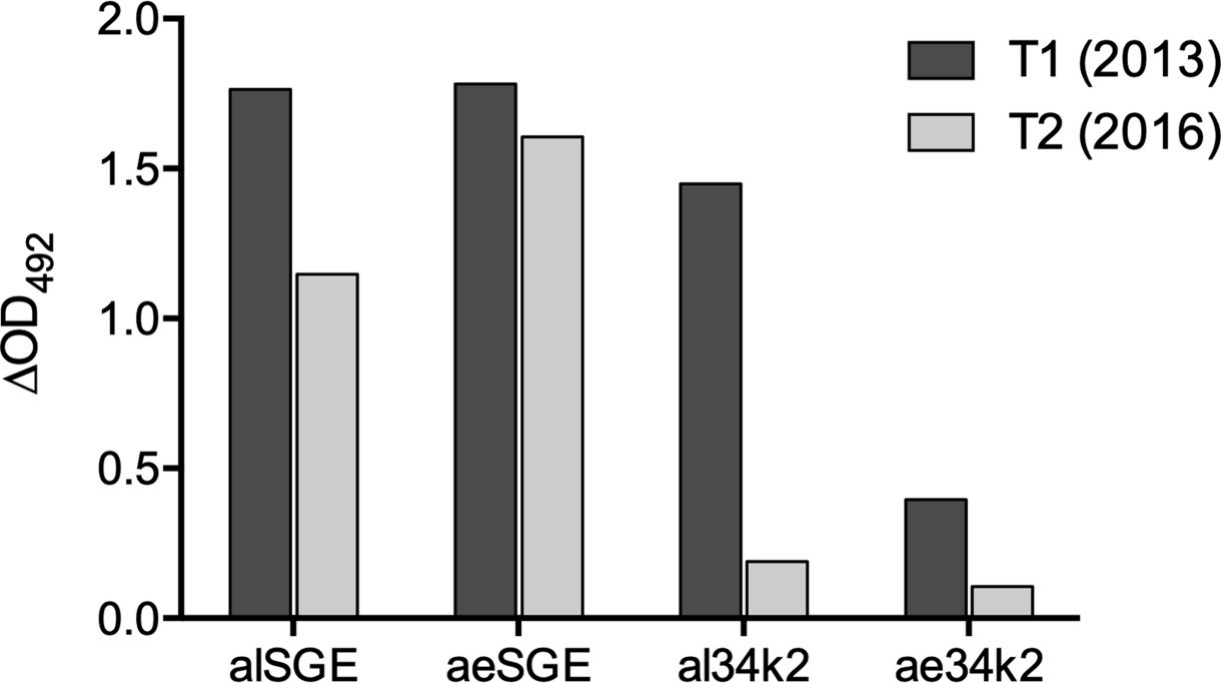
Levels of anti-SGE and anti-34k2 IgG in a human hyperimmune serum. Levels of IgG antibodies directed against alSGE, aeSGE, al34k2 and ae34k2 were determined at two different time points: (i) T1 (2013), shortly after regularly feeding an *Ae*. *albopictus* colony approximately every two weeks for 4 months; (ii) T2 (2016), after the donor had not been feeding *Ae*. *albopictus* nor other *Aedes* spp colonies for at least two years.

The availability of a single human serum hyperimmune to *Ae*. *albopictus* saliva offered the opportunity to obtain some preliminary indication on the immunogenicity to humans of al34k2, and eventually on the immune cross-reactivity to ae34k2. The human serum was obtained from a donor at two different time points: (i) in February 2013 (T1), after feeding for a period of approximately four months an *Ae*. *albopictus* colony, and (ii) in May 2016 (T2), after the volunteer had not been feeding *Ae*. *albopictus* nor other *Aedes* spp colonies for at least two years (and had, likely, only natural exposure to *Aedes* mosquitoes). An intense IgG response against both alSGE and aeSGE was detectable at T1, confirming the hyperimmunization of the donor against *Ae*. *albopictus* saliva and displaying a wide IgG cross-reactivity to SGE from *Ae*. *aegypti*. On the contrary, the IgG response to al34k2 appeared considerably higher as compared to the response to ae34k2 (Fig 4). At the time point T2 the IgG response to both alSGE and aeSGE persisted, even though at a slightly lower level. On the contrary, the specific IgG response to al34k2 had a remarkable decrease and also levels of anti-ae34k2 IgG appeared reduced. Overall, despite the obvious intrinsic limitations due to the availability of a single human serum and by the hyperimmune status, these observations suggest that al34k2 is immunogenic to humans and that, as observed in mice, there may be a limited cross-reactivity to the two orthologous 34k2 proteins.

## Discussion

Toward the development of immunoassays based on vector salivary proteins to assess human exposure to *Aedes* mosquitoes, we report here the use of a murine model to test candidate peptides and recombinant salivary proteins from *Ae*. *albopictus* and *Ae*. *aegypti*. The choice of an experimental animal model, despite intrinsic limitations as the need for downstream validation in humans, has the advantage to allow the setting of strictly controlled conditions of exposure and, possibly, to provide valuable information on the kinetics of mounting/decay of the humoral response and eventually on its species-specificity. The regimen of mice exposure was previously successfully used for mice immunization by exposure to anopheline mosquitoes [39, 40] and the candidate salivary proteins analyzed in our study were already known to be culicine-specific, absent in the saliva of anopheline mosquitoes and not encoded in their genomes [24, 25]. The protocol of exposure was effective for both *Ae*. *albopictus* and *Ae*. *aegypti*: in both cases all exposed mice, with some individual variation, carried in their sera relatively high levels of IgG antibodies against SGE from the same species. The anti-SGE IgG response could be detected in most mice already after the second exposure but the peak was reached only after the fourth and last exposure. Although the absence of later time points does not allow to clarify in more detail the kinetics, the mice anti-saliva IgG responses were essentially stable up to 3 months later, with some apparent decline in half of the immunized mice 5 months after the end of exposure (Fig 1A and 1B). Although several studies investigated the effects of mosquito saliva on host immune cells and/or on arboviral transmission, to our knowledge a detailed analysis of development and decay of mice IgG responses to *Aedes* saliva or salivary proteins has not been previously performed. However, it is pretty well established that in conditions of natural exposure the human IgG antibody response against mosquito saliva progressively declines after termination or drastic reduction of the exposure, even though the specific timing may depend by several factors such as age and history (intensity and persistence) of exposure. As far as *Aedes* mosquitoes are concerned, a decreased IgG antibody response against *Ae*. *aegypti* saliva was reported in French soldiers three months after their return from a travel period in tropical Africa [60], and similar results were obtained in Colombians coming back to an *Ae*. *aegypti*-free area after travelling to DENV endemic areas [61]. Also, a significant reduction of IgG levels against *Ae*. *albopictus* saliva was found already six weeks after the implementation of vector control interventions in La Reunion Island [62]. A decrease in the response against alSGE was also observed in the hyperimmune donor between time points T1 and T2, although high anti-alSGE IgG levels still persisted at T2, that is three years after feeding an *Ae*. *albopictus* colony (Fig 4). This very long-lived anti-saliva response observed in the donor it is possibly the result of the hyperimmunization combined to the likely natural exposure to *Ae*. *albopictus*, a species widely distributed in Italy, the country of residence of the donor at that times.

We also measured the IgG response to alSGE in *Ae*. *aegypti*-exposed mice (and vice versa), as well as the response to aeSGE of the donor hyperimmune to *Ae*. *albopictus* saliva. In principle, immune cross-reactivity between responses induced by salivary secretions of the two *Aedes* species it is not surprising considering the wide overlap and degree of conservation of their salivary protein repertoires [41, 42]; indeed, all mice exposed to one *Aedes* species also responded to SGE from the other species (Fig 1C and 1D). The small number of experimental mice, the unexpectedly lower feeding efficiency of *Ae*. *albopictus* as compared to *Ae*. *aegypti* (21 vs 28 mosquitoes/mice/exposure) and the slightly higher protein content of aeSGE versus alSGE (10 SG/ml = 11.0 μg/ml vs 8.6 μg/ml, respectively) preclude any reliable quantitative evaluation of immune cross-reactivity. Nevertheless, a tentative provisional evaluation (see Methods) suggests a level of approximately 50% cross-reactivity in our experimental mice. A high level of immune cross-reactivity was also observed in the hyperimmune donor, who showed an almost identical response to SGEs from the two *Aedes* species (Fig 4). While no previous data on mice exposed to these two mosquito species are available for comparison, on the contrary, low level of immune cross-reactivity between *Ae*. *albopictus* and *Ae*. *aegypti* SGEs has been previously reported in humans in conditions of natural exposure. This was the case for individuals from Reunion Island, who were only exposed to *Ae*. *albopictus*, as compared to individuals from Bolivia, only exposed to *Ae*. *aegypti* [63]. A likely interpretation of this apparent discrepancy is that the level of immune cross-reactivity is dependent on the intensity of immunization, history of exposure and responder status. This would be in agreement both with the observations of Doucoure and collaborators, who found high levels of cross-reactivity among individuals with high anti-saliva IgG levels [63], and with the results reported here, where both the immunized mice and the human volunteer can be considered as intensely exposed and high responders. Overall, besides the already mentioned limitations (small number of mice, single human serum, hyperimmunization) and the apparent discrepancies, the IgG responses to SGE clearly point to the effective immunization of both mice and human donor against *Aedes* saliva, which allowed to proceed to the main experimental aim of our study, that is testing the immunogenicity of candidate peptides and recombinant proteins.

In general, both peptides and full length recombinant proteins are widely used in immunoassays with relative advantages and disadvantages. Recombinant proteins, carrying the conformational epitopes typical of the native forms, may provide higher sensitivity but, on the other side, their expression/purification can be difficult, time consuming and less reproducible. Peptides, instead, can be commercially obtained by chemical synthesis with very good reproducibility and are easier to be used in the field. Moreover, they have been already successfully employed to assess human exposure to mosquitoes, as testified by gSG6-P1 for malaria vectors [27, 52–54] and the Nterm-34kDa salivary peptide for *Aedes* vectors [35, 38]. Guided by B-cell epitope prediction software, we designed five peptides on the culicine-specific 34k1, 34k2 and 62k1 *Ae*. *albopictus* salivary proteins. The peptides were designed on the N- and/or C-terminal protein regions, which are more likely to be exposed on protein surfaces and therefore visible to the immune system; this was certainly the case for alb34k2-P2 and alb34k2-P3, as indicated by mapping the peptides on the crystal structure of the *Ae*. *albopictus* 34k2 protein (P. Gabrieli and F. Forneris, personal communication). However, none of these peptides appeared immunogenic to mice, and this was especially surprising for alb34k1-P1 which is the orthologue of Nterm-34kDa [35], although the two peptides show a certain degree of divergency, since they share 12 over 19 amino acid residues with a three amino acid insertion in the *Ae*. *albopictus* protein. We cannot provide a clear and convincing explanation for this failure: perhaps the fact that we did not use for peptide design also T-cell epitope prediction software as in previous selection strategies [27, 35] may have contributed, or there may be some other technical reason that we could not identify despite the several efforts. Nevertheless, we report here these negative results because we believe this may represent anyway a useful information for others working in the field and, overall, we should point out that, although these peptides appeared not antigenic to mice, no conclusions can be drawn concerning their potential antigenicity to humans.

The main finding of our study is certainly linked to the specific IgG responses to the al34k2 and ae34k2 recombinant proteins observed in immunized mice and in the single human hyperimmune donor. Two out of the four *Ae*. *albopictus*-immunized mice developed anti-al34k2 IgG antibodies: the response was evident only after the fourth/last exposure and stayed essentially unchanged in both mice up to two months (+60) after the end of exposure. Afterwards, IgG levels appeared to decrease in M10 and persisted at high level in M12 (Fig 3A). The other two mice (M9 and M11) did not show IgG responses to al34k2 at any time point. This may be due to the less effective immunization of these mice, who also showed lower IgG responses to alSGE as compared to M10 and M12, or perhaps to a limiting antigenicity of the al34k2 salivary protein. It may be also possible that these mice presented specific IgM but no IgG. The available data do not allow to sort this out, however, it should be emphasized that high individual variability, both in the quality and in the intensity of the host response to mosquito salivary antigens, has been repeatedly observed in many different studies, as exemplified by the IgG responses to the *An*. *gambiae* salivary proteins gSG6 and cE5 measured in the same individuals highly exposed to malaria vector bites in a hyperendemic area of Burkina Faso [29]. Therefore, despite the absence of IgG responses in M9 and M11, and also considering the small total number of mice analyzed, we believe that these observations provide preliminary but promising indications on the antigenicity of al34k2. As far as the *Ae*. *aegypti*-exposed mice are concerned, they all exhibited an intense IgG response to ae34k2 clearly pointing out its immunogenicity too. Also in this case the response appeared only after the fourth/last exposure in three of the four mice (M5, M6 and M8); afterwards, it stayed essentially unchanged up to 5 months later in M5 and M8, while appeared to gradually decrease in M6. In the remaining mice (M7) the IgG response exhibited a somewhat different kinetic with a later appearing, moderate growth up to 3 months (+90) and then a slight decrease (Fig 3B). Overall, we can conclude that both al34k2 and ae34k2 were immunogenic to mice even though, considering the number of mice showing detectable IgG responses and their intensities, the latter seems to evoke stronger IgG antibody responses. However, it should be kept in mind that the unexpected lower feeding efficiency of *Ae*. *albopictus* (21 bites/mouse/exposure) as compared to *Ae*. *aegypti* (28 bites/mouse/exposure), and the possible resulting less effective immunization, may also account at least in part for the observed difference. Strikingly, despite the relatively high degree of identity (62%) between the two orthologous proteins, we observed no immune cross-reactivity in the exposed mice: no anti-ae34k2 IgG antibodies were detectable at any time point in the mice exposed to *Ae*. *albopictus*, and vice versa for al34k2 and *Ae*. *aegypti*-exposed mice. This observation is intriguing since it may represent the basis for the development of species-specific assays to assess host exposure to *Ae*. *albopictus* or *Ae*. *aegypti*, respectively. Indeed, while in principle the availability of a single marker allowing for the simultaneous evaluation of host exposure to *Aedes* species may be desirable and practical, in same epidemiological settings species-specific assays may prove very useful. For example, considering their different importance in arboviral transmission, this may be the case in areas where these two species coexist or also in areas where just one species is present but in sympatry with other *Aedes* species of low or no relevance for arboviral transmission.

The availability of serum from a donor hyperimmune to *Ae*. *albopictus* saliva allowed to get some preliminary insights into the human IgG response to al34k2. Confirming previous evidence obtained by 2D-immunoblot analysis of *Ae*. *albopictus* SGE [55], al34k2 also appeared strongly immunogenic to humans: an intense IgG response to al34k2 was evident at time point T1, shortly after the donor had fed an *Ae*. *albopictus* colony. The same donor, at the same time point, showed an IgG response of much lower intensity (~27%) to the *Ae*. *aegypti* ae34k2, suggesting limited immune cross-reactivity in humans (Fig 4). Noteworthy, while the IgG responses to al34k2 showed a marked decrease at time point T2, the antibody responses to SGE persisted at a much higher extent. As already mentioned, previous studies in conditions of natural exposure indicated that human IgG responses to *Aedes* saliva are short-lived [60–62]. The long persistence of the anti-saliva response in the hyperimmune donor may be the results of the hyperimmunization and of the likely natural exposure of the donor to bites of *Ae*. *albopictus*; moreover, the high inter-individual variability of the anti-saliva response, even in condition of natural exposure, should be kept in mind [60–62]. On the other side, the decay of the anti-al34k2 IgG responses is not surprising considering that mosquito saliva is a complex mixture of hundred or more proteins, and that host antibody response to these proteins is heterogenous, with some eliciting short-lived IgG responses and others triggering longer-lasting antibody responses [29]. In this respect, it is important to clarify that a critical property for a good serological marker of host exposure to vectors is the duration of the IgG antibody response. An ideal marker should evoke a short-term response, allowing for the detection of spatial and temporal variations in host exposure: this is the case for the *An*. *gambiae* gSG6 protein or the gSG6-P1 [26–33, 51, 54] and for the *Ae*. *aegypti* Nterm34kDa peptide [34–38]. In our study the immunized mice showed a persistence of the IgG responses to al34k2 and ae34k2 for at least three months after the end of exposure, with some trend to decrease afterwards, whereas the single human serum analyzed here cannot provide any useful information about timing. Further analyses in humans will be crucial to better understand the kinetics of decay of anti-al34k2 and ae34k2 IgG responses in condition of natural exposure and clarify their suitability as serological tools to detect seasonal variations of host exposure to *Aedes* mosquitoes.

In conclusion, we would like to point out that the Nterm34kDa peptide, the best tool presently available for the serological assessment of human exposure to *Aedes* mosquito vectors, is designed on the 34k1 salivary protein of *Ae*. *aegypti* and that 34k1 and 34k2 proteins are largely divergent: they only share 32–33% amino acid identity in *Ae*. *albopictus* and *Ae*. *aegypti*, respectively. This implies that the 34k2 proteins, whose expression, purification and testing are reported here, represent real novel candidates. Both the mice and human samples analyzed in our study provided some preliminary but certainly encouraging information on the antigenicity of the al34k2 and ae34k2 proteins and on the IgG response they evoke in exposed hosts; however, it should be kept in mind that this information refers to a small number of mice and a single human donor in conditions of intense and repeated exposure. Measurements of the humoral response in relatively large group of individuals naturally subjected to *Aedes* bites from locations with different mosquito densities and/or from the same area in different seasons (high and low *Aedes* density) will be essential to get a clearer idea on their suitability as reliable antigens to detect spatial and temporal variations of human exposure to *Ae*. *albopictus* and/or *Ae*. *aegypti*.

## Supporting information

S1 Table Number of *Ae*. *albopictus* and *Ae*. *aegypti* mosquitoes who successfully fed on experimental mice following each exposure. (PDF)
