# Supplementary material for: Analysis in a murine model points to IgG responses against the 34k2 salivary proteins from Aedes albopictus and Aedes aegypti as novel promising candidate markers of host exposure to Aedes mosquitoes
Source: PLoS Negl Trop Dis. 2019 Oct 16;13(10):e0007806. doi: 10.1371/journal.pntd.0007806 (PMC6816578; doi:10.1371/journal.pntd.0007806)
Supplement: S1 Table — (PDF) [file pntd.0007806.s001.pdf]

Table S1. Mice immunization by exposure to *Ae. aegypti* or *Ae. albopictus* bites.

	Ae. aegypti										***Ae. albopictus***									
	**M5**		**M6**		**M7**		**M8**		**mean/exposure**		**M9**		**M10**		**M11**		**M12**		**mean/exposure**	
	fed (n)	fed (%)	fed (n)	fed (%)	fed (n)	fed (%)	fed (n)	fed (%)	fed (n)	fed (%)	fed (n)	fed (%)	fed (n)	fed (%)	fed (n)	fed (%)	fed (n)	fed (%)	fed (n)	fed (%)
Exposure 1	30	81.1	27	64.3	27	84.4	23	76.7	26.75	76.6	23	54.8	23	65.7	20	47.6	23	76.7	22.25	61.2
Exposure 2	34	89.5	35	89.7	40	81.6	40	72.7	37.25	83.4	23	40.4	23	40.4	16	27.1	28	44.4	22.5	38.1
Exposure 3	34	97.1	21	75.0	25	86.2	27	77.1	26.75	83.9	24	66.7	13	41.9	16	59.3	20	62.5	18.25	57.6
Exposure 4	23	79.3	19	82.6	18	81.8	21	70.0	20.25	78.4	26	49.1	14	32.6	19	36.5	21	47.7	20	41.5
total	121		102		110		111		111		96		73		71		92		92	
**mean/mouse**	30.3	86.8	25.5	77.9	27.5	83.5	27.8	74.1	27.8	80.6	24.0	52.7	18.3	45.1	17.8	42.6	23.0	57.8	20.8	49.6

The number of mosquitoes fed and percentages for each individual mouse during the four exposures are shown.
The mean number and the mean percentage of mosquitoes fed/mouse/exposure are highlighted.
